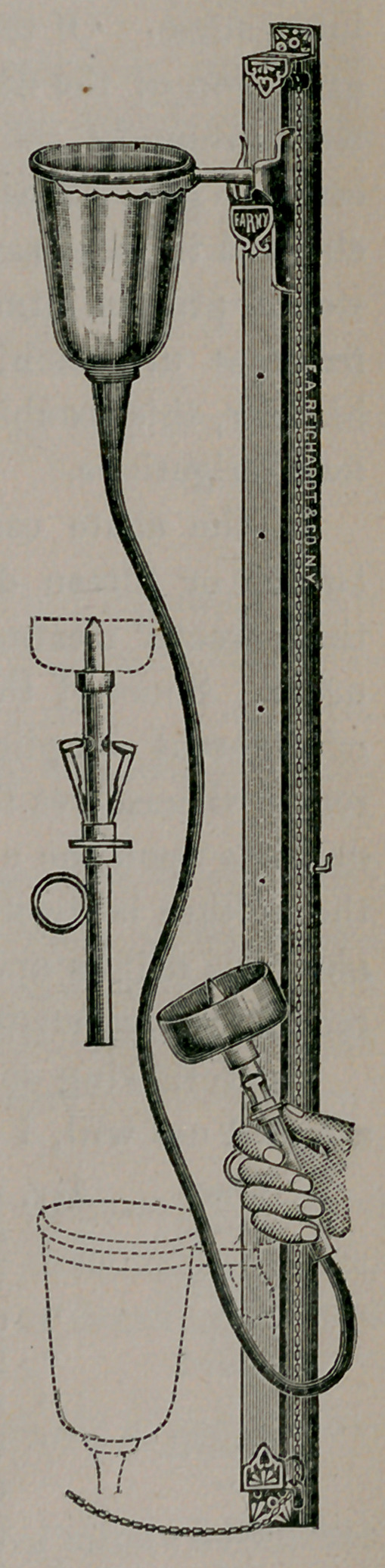# The Treatment of Gonorrhœa by Hydrostatic Irrigation*Read before Atlanta Society of Medicine, January 7, 1898.

**Published:** 1898-02

**Authors:** W. L. Champion

**Affiliations:** Atlanta, Ga.; President Atlanta Society of Medicine


### THE TREATMENT OF GONORRHŒA BY HYDROSTATIC IRRIGATION.*

By W. L. CHAMPION, M.D., Atlanta, Ga.
President Atlanta Society of Medicine.

I have here a few notes to read, and then I wish to demonstrate a method which I think is far superior to any other for the treatment of urethral and vesical diseases.

It is conceded by all that gonorrhœa is a very unsatisfactory disease to treat. The reason of this is the physician allows the patient to treat himself. The patient usually abandons treatment as soon as the discharge is checked, which we all know is not proof that the disease is cured. We should always instruct our patients that so long as there are clap-shreds in the urine the urethra is diseased, and treatment should not be discontinued. Neglect in this particular is frequently the cause of a single attack covering a period of months and sometimes years.

I hold that every case of gonorrhœa should be treated by the physician, when it is possible for the patient to make regular visits

*Read before Atlanta Society of Medicine, January 7, 1898.

to the office. The patient should be given nothing to take internally, nor any injection to use.

The method which I wish to demonstrate to-night was first suggested by Professor Janet of Paris, and the apparatus I use is one devised by Dr. Valentine of New York. Prior to July, 1896, I used the irrigation method with a soft rubber catheter, instead of a nozzle as you see attached to this instrument. The drug I have found most satisfactory is permanganate of potash. Nitrate of silver, bichloride mercury, boric acid, and other drugs are used as indicated. The container, which holds about a quart of the solution to be used, is placed eight or nine feet from the floor. The glans penis is first cleansed, then the anterior urethra is thoroughly washed out, then the nozzle is pressed gently against the meatus and the patient told to breathe deeply or try to urinate, and the fluid will flow back into the bladder; when the bladder commences to feel full the patient is allowed to pass the fluid out, and the bladder is refilled. By this means the urethra is distended to its full capacity, which forces the pus and germs from the glands and follicles of the canal.

So long as the urethra is very sensitive or highly inflamed I only irrigate the anterior portion, unless there are indications of extension of the disease into the deep urethra or bladder.

Treatment is commenced as soon as the patient presents himself, and the discharge is stopped usually within four or five days; the redness, pain, and swelling commences to subside and the course of the disease is cut short. So instead of having a gonorrhœa to treat for weeks and sometimes months, the course of the disease is limited to twelve days or two or three weeks.

I have a record of one hundred and fifty-three cases treated in my private practice by this method. Sixty per cent. were acute cases of gonorrhœa, thirty per cent. were chronic cases of gonorrhœa, and ten per cent. cases of cystitis. Of this number only one case developed epididymitis, and this was produced, I think, by sexual intercourse. Of the cases of gonorrhœa only two cases showed any extension of the disease into the deep urethra or bladder, and these were promptly relieved by continuance of the treatment. In all cases of gonorrhœa, whether acute or chronic, the discharge was checked in from seventy-two to ninety-six hours, when the patient would present himself regularly for treatment. In all cases of frequent urination, due to irritation in deep urethra or neck of bladder, this troublesome symptom was relieved with from two to four irrigations.

In the acute cases of gonorrhœa a cure was perfected within twelve or fifteen days in eleven cases, but the majority required three weeks' treatment before all clap-shreds disappeared from the urine. Some of the cases required even a longer time on account of different complications that were present when the patients presented themselves for treatment. The duration of a single attack depends upon the number of attacks the patient has had, whether the urethra is strictured or not, whether the patient abstains from alcoholic drinks and sexual intercourse, and presents himself as directed for treatment.

The following formulary of a solution of permanganate of potash as I use will, I think, give general satisfaction.

| | | | | | |
|---|---|---|---|---|---|
| First | day, | first visit, | anterior | irrigation | 1–6000 |
| " | " | 6 p m., | " | " | 1–6000 |
| Second | " | 9 a.m., | " | " | 1–4000 |
| " | " | 6 p.m., | " | " | 1–3000 |
| Third | " | 9 a.m., | " | " | 1-3000 |
| " | " | 6 p.m., | " | " | 1–3000 |
| Fourth | " | 9 a.m., | " | " | 1–3000 |
| " | " | 6 p.m., | " | " | 1–3000 |
| Fifth | " | 9 a.m., | " | " | 1–3000 |
| " | " | 6 p.m., | " | " | 1–2000 |
| Sixth | " | 9 a.m., | intravesical | irrigation | 1–6000 |
| " | " | 6 p.m., | anterior | " | 1–4000 |
| Seventh | " | 9 a.m., | intravesical | " | 1–6000 |

| | | | | | |
|---|---|---|---|---|---|
| Eighth | day, 9 a.m., | intravesical irrigation | | .......... | 1–4000 |
| Ninth | " 9 a.m, | " | " | .......... | 1–4000 |
| Tenth | " 9 a.m., | " | " | .......... | 1–4000 |
| Eleventh | " 9 a.m., | " | " | .......... | 1–3000 |
| Twelfth | " 9 a.m., | " | " | .......... | 1–2000 |
| Thirteenth | " 9 a.m., | " | " | .......... | 1–2000 |
| Fourteenth | " 9 a.m., | " | " | .......... | 1–2000 |
| Fifteenth | " 9 a.m., | " | " | .......... | 1-1000 |

[NOTE.—The apparatus described and pictured is made by Messrs. Alfred Reichardt & Co., of 27 Barclay St., New York, to whom we are indebted for the cut on preceding page.]

## Figures and Tables

**Figure f1:**